# Salicylic acid alleviates decreases in photosynthesis under heat stress and accelerates recovery in grapevine leaves

**DOI:** 10.1186/1471-2229-10-34

**Published:** 2010-02-23

**Authors:** Li-Jun Wang, Ling Fan, Wayne Loescher, Wei Duan, Guo-Jie Liu, Jian-Shan Cheng, Hai-Bo Luo, Shao-Hua Li

**Affiliations:** 1 Institute of Botany, Chinese Academy of Sciences, Beijing, 100093, PR China; 2 College of Agriculture and Biology Technology, China Agricultural University, Beijing 100093, PR China; 3 College of Agriculture and Natural Resources, Michigan State University, East Lansing, 48824, MI, USA; 4 Key Laboratory of Pant Germplasm Enhancement and Speciality Agriculture, Wuhan Botanical Garden, Chinese Academy of Sciences, Wuhan 430074, PR China

## Abstract

**Background:**

Although the effect of salicylic acid (SA) on photosynthesis of plants including grapevines has been investigated, very little is yet known about the effects of SA on carbon assimilation and several components of PSII electron transport (donor side, reaction center and acceptor side). In this study, the impact of SA pretreatment on photosynthesis was evaluated in the leaves of young grapevines before heat stress (25°C), during heat stress (43°C for 5 h), and through the following recovery period (25°C). Photosynthetic measures included gas exchange parameters, PSII electron transport, energy dissipation, and Rubisco activation state. The levels of heat shock proteins (HSPs) in the chloroplast were also investigated.

**Results:**

SA did not significantly (*P *< 0.05) influence the net photosynthesis rate (*P*_n_) of leaves before heat stress. But, SA did alleviate declines in *P*_n _and Rubisco activition state, and did not alter negative changes in PSII parameters (donor side, acceptor side and reaction center Q_A_) under heat stress. Following heat treatment, the recovery of *P*_n _in SA-treated leaves was accelerated compared with the control (H_2_O-treated) leaves, and, donor and acceptor parameters of PSII in SA-treated leaves recovered to normal levels more rapidly than in the controls. Rubisco, however, was not significantly (*P *< 0.05) influenced by SA. Before heat stress, SA did not affect level of HSP 21, but the HSP21 immune signal increased in both SA-treated and control leaves during heat stress. During the recovery period, HSP21 levels remained high through the end of the experiment in the SA-treated leaves, but decreased in controls.

**Conclusion:**

SA pretreatment alleviated the heat stress induced decrease in *P*_n _mainly through maintaining higher Rubisco activition state, and it accelerated the recovery of *P*_n _mainly through effects on PSII function. These effects of SA may be related in part to enhanced levels of HSP21.

## Background

Heat stress due to high ambient temperatures is a serious threat to crop production [1]. Photosynthesis is one of the most sensitive physiological processes to heat stress in green plants [2]. Photochemical reactions in thylakoid lamellae in the chloroplast stroma have been suggested as the primary sites of injury at high temperature [3]. Heat stress may lead to the dissociation of the oxygen evolving complex (OEC), resulting in an imbalance during the electron flow from OEC toward the acceptor side of photosystem II (PSII) [4]. Heat stress may also impair other parts of the reaction center, e.g., the D1 and/or the D2 proteins [5]. Several studies have suggested that heat stress inhibits electron transport at the acceptor side of PSII [6-8]. Direct measurements of the redox potential of Q_A _have demonstrated that heat stress induces an increase in the midpoint redox potential of the Q_A_/Q_A_^- ^couple in which electron transfer from Q_A_^- ^to the secondary quinone electron acceptor of PSII (Q_B_) is inhibited [6-8]. On the other hand, some studies have shown that the decreased photosynthesis could be attributed to the perturbations of biochemical processes, such as decreases in ribulose bisphosphate carboxylase/oxygenase (Rubisco) activity and decreases in ribulose-1,5-bisphosphate (RuBP) or Pi regeneration capacity [9].

Plants have evolved a series of mechanisms to protect the photosynthetic apparatus against damage resulting from heat stress. For example, many studies have shown that heat dissipation of excess excitation energy is an important mechanism [10,11]. When plants are subjected to heat stress, a small heat shock protein is expressed that binds to thylakoid membranes and protects PSII and whole-chain electron transport [12]. But, when plants are subjected to more severe stress, these protective mechanisms may be inadequate. However, some growth regulators have been used to induce or enhance these protective functions [13,14].

Salicylic acid (SA) is a common plant-produced phenolic compound that can function as a plant growth regulator. Various physiological and biochemical functions of SA in plants have been reported [15], and SA has received much attention due to its role in plant responses to abiotic stresses, including heat stress. SA application may improve photosynthetic capacity in spring wheat and barley under salt stress and drought stress [16,17] and *Phillyrea angustifolia *and wheat seedlings under drought stress [18,19]. But, relatively little is yet known about SA-related mechanisms that alleviate the decline of photosynthesis in these studies. In addition, exogenous application of SA or acetylsalicylate has been shown to enhance thermotolerance in tobacco and *Arabidopsis *[20-24]. Wang and Li [25] reported that spraying with a 0.1 mM solution of SA decreased thiobarbituric acid-reactive substances and relative electrolyte leakage in young grape leaves under heat stress, indicating that SA can induce intrinsic heat tolerance in grapevines. Dat et al. [20] showed that thermotolerance (expressed as survival rate after heat treatment) of mustard (*Sinapis alba *L.) seedlings could be obtained by SA treatment. Lopez-Delgado et al. [22] reported that thermotolerance (expressed as survival rate after heat treatment) can be induced in potato microplant tissues by treatment with acetylsalicylic acid, and Wang et al. [26] reported that SA treatment can maintain at higher *P*_n _in grape leaves under heat stress. There are, however, very few reports on how SA affects the photochemical aspects of PSII in plants under heat stress, such as energy absorption, utilization, and dissipation of excess energy.

Worldwide, grape has become one of the most productive and important specialty crops. In many production regions, the maximum midday air temperature can reach more than 40°C, which is especially critical at veraison when the berries are rapidly accumulating photosynthates. Climate change may produce more frequent high temperature conditions close to the current northern limit of grape cultivation [27-29]. Extreme temperatures may endanger berry quality and economic returns [30]. Wang and Li [25] have previously reported that SA alleviates heat damage of plants by up-regulating the antioxidant system. Here, in the present experiment, we investigated the effect of SA on photosynthesis of grape leaves before, during and after heat stress.

## Results

### Net photosynthesis rate (*P*_n_), substomatal CO_2 _concentration (*C*_i_) and stomatal conductance (*g*_s_)

**Figure 1:**
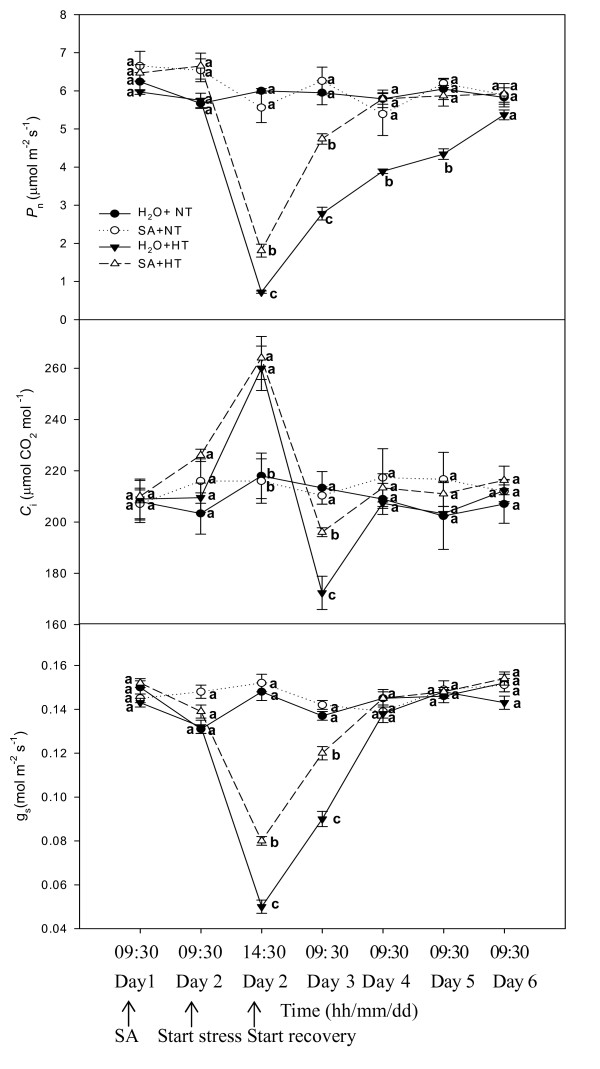
***P*_n_, *C*_i_ and *g*_s_ in leaves of grape plants sprayed with H_2_O (*filled circles*) and SA (*open circles*) at normal growth temperature (NT, 25°C), and treated with H_2_O (*filled triangles*) and SA (*open triangles*) under heat stress (HT, 43°C) and recovery**. Each value is the mean ± SE of 4 replicates. 0.1 mM SA solution or H_2_O was sprayed at 9:30 h on Day 1, immediately afterwards photosynthesis and chlorophyll fluorescence parameters were measured. Heat stress was from 9:30 to 14:30 h on Day 2. The recovery period was from 14:30 h on Day 2 to 9:30 h on Day 6. At the same time point, numerical values with different letters are significantly different (*P *< 0.05).

At normal growth temperature, spraying SA did not induce significant (*P *< 0.05) changes in *P*_n_, *C*_i _and *g*_s _in the grapevines (Fig. 1). When these plants were heat stressed at 43°C for 5 h, *P*_n _and *g*_s_sharply declined while *C*_i _abruptly rose; however, the SA-treated plants had significantly higher *P*_n _values than the controls (H_2_O + HT). There was no significant difference in *C*_i _between SA-treated and control plants in normal growth conditions. During recovery, *P*_n _and *g*_s _of heat treated plants increased and *C*_i _steeply decreased (on Day 3). *P*_n_, *C*_i _and *g*_s _of these plants then gradually increased, and the SA-treated plants had higher *P*_n _than the control plants. However, no significant differences were found in *P*_n_, *C*_i _and *g*_s _between SA and control plants on Day 6 (Fig. 1).

### Donor side, reaction centre and acceptor side of PSII

**Figure 2:**
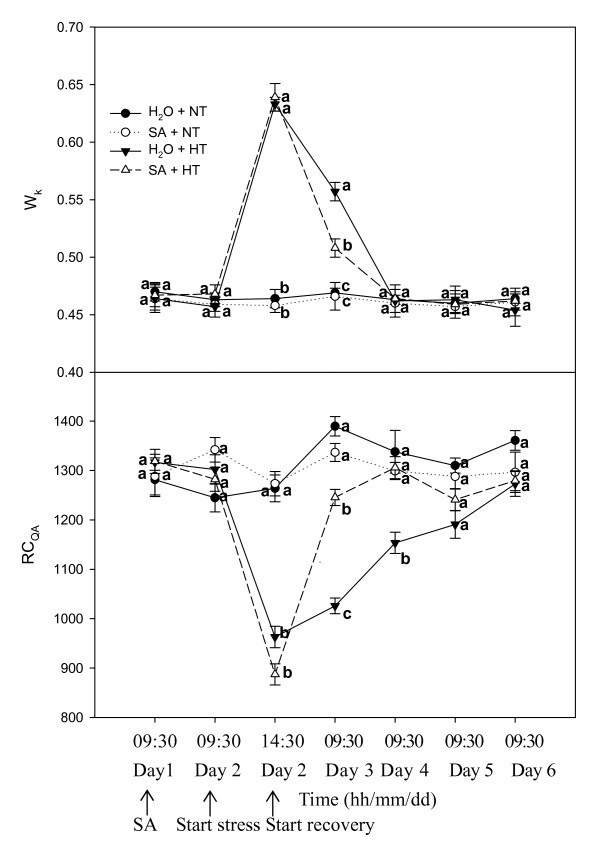
**Donor side parameter (W_K_) and reaction center parameter (RC_QA_) of PSII in leaves of grape plants sprayed with H_2_O (*filled circles*) and SA (*open circles*) under normal growth temperature (NT, 25°C), and treated with H_2_O (*filled triangles*) and SA (*open triangles*) under heat stress (HT, 43°C) and recovery**. Each value is the mean ± SE of 4 replicates. Treatment conditions are described in Fig. 1. At the same time point, numerical values with different letters are significantly different (*P *< 0.05).

In general, a typical polyphasic rise of fluorescence transients determined by a Handy Plant Efficiency Analyzer (Hanstech, UK) includes phases O, J, I and P. It has been shown that heat stress can induce a rapid rise in these polyphasic fluorescence transients. This rapid rise, occurring at around 300 μs, has been labeled as K, and is the fastest phase observed in the OJIP transient which, consequently, becomes an OKJIP transient [31]. It has also been shown that phase K is caused by an inhibition of electron transfer to the secondary electron donor of PSII, Yz, which is due to a damaged oxygen evolving complex (OEC). The amplitude of step K can therefore be used as a specific indicator of damage to the OEC [32]. Fig. 2 shows the changes in amplitude in the K step expressed as the ratio W_K_. SA spraying did not result in obvious changes of W_K _in grape leaves under normal temperature. When control and SA-sprayed plants were stressed by heat, W_K _of both went up quickly, and similarly. During recovery W_K _of the SA treatment dropped more quickly than W_K _of the control. Moreover, W_K _of the SA treatment was significantly lower than that of the control on the first day of recovery (Day 3).

The density of RC_QA _in the control and SA-treated leaves was unchanged at normal temperature. When heat stress was imposed, density of RC_QA _declined rapidly. During the recovery period, density of RC_QA _of SA-sprayed leaves rose and nearly reached normal levels on Day 3, but the control RC_QA _recovered slowly, and reached normal levels on Day 5 (Fig.2).

**Figure 3:**
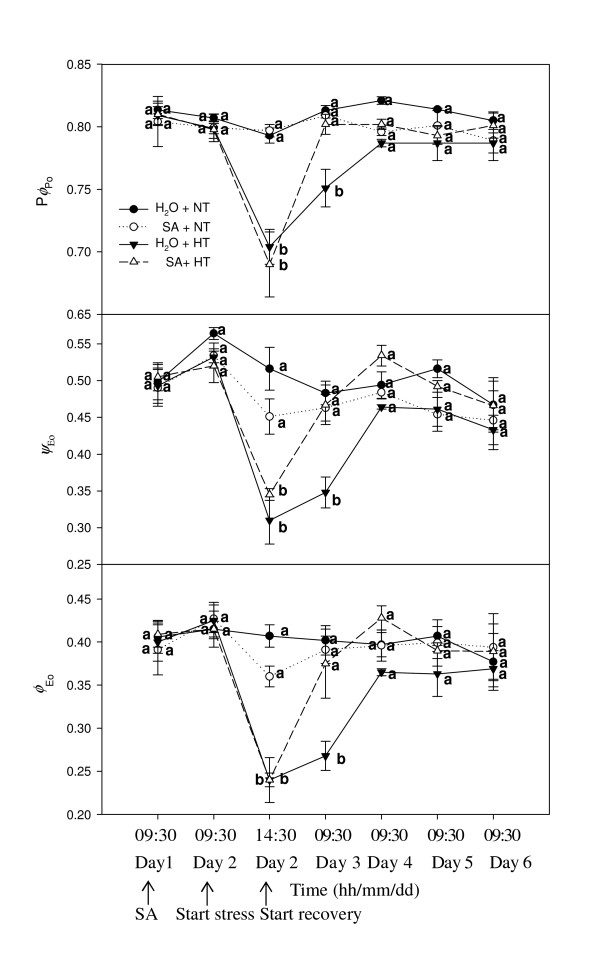
***ϕ*_Po_ and acceptor parameters (*ψ*_Eo_ and Φ_Eo_) in leaves of grape plants sprayed with H_2_O (*filled circles*) and SA (*open circles*) at normal growth temperature (NT, 25°C), and treated with H_2_O (*filled triangles*) and SA (*open triangles*) under heat stress (HT, 43°C) and recovery**. Each value is the mean ± SE of 4 replicates. Treatment conditions are described in Fig. 1. At the same time point, numerical values with different letters are significantly different (*P *< 0.05).

Fig. 3 demonstrates (1) the changes in maximum quantum yield for primary photochemistry (*ϕ*_Po_), (2) the efficiency with which a trapped excitation can move an electron into the electron transport chain further than Q_A_^- ^(*ψ*_Eo_), and (3) the quantum yield of electron transport (*ϕ*_Eo_) in grape leaves. Under normal temperatures, spraying SA did not change *ϕ*_Po_, *ψ*_Eo _and *ϕ*_Eo_. With heat stress, *ϕ*_Po_, *ψ*_Eo _and *ϕ*_Eo _in both SA-treatedand control leaves significantly declined. During recovery, *ϕ*_Po_, *ψ*_Eo _and *ϕ*_Eo _of SA-treated leaves rapidly increased, and these parameters were markedly greater in SA-treated leaves than in the controls on Day 3.

**Figure 4:**
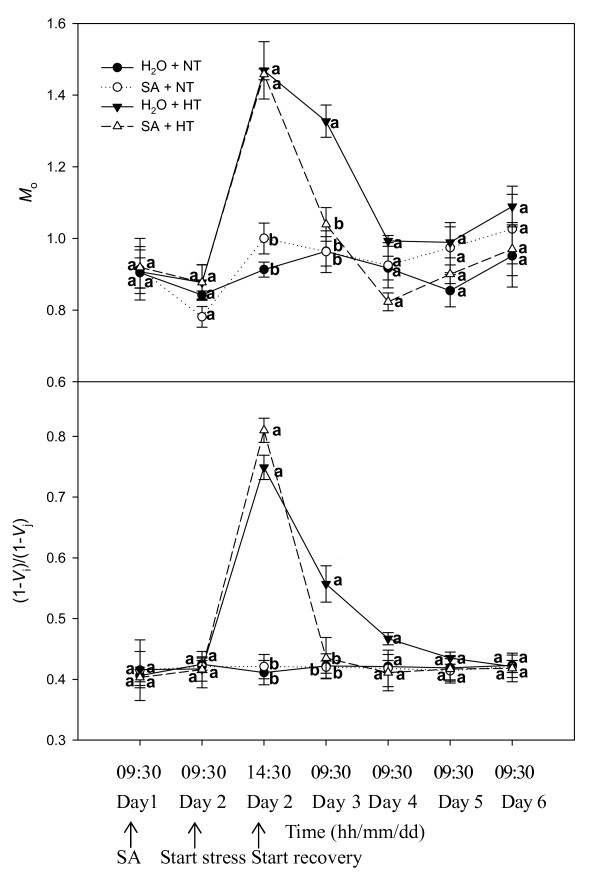
**Acceptor sides parameters *M*_o _and (1-*V*_i_)/(1-*V*_j_) in leaves of grape plants sprayed with H_2_O (*filled circles*) and SA (*open circles*) at normal growth temperature (NT, 25°C), and treated with H_2_O (*filled triangles*) and SA (*open triangles*) under heat stress (HT, 43°C) and recovery**. Each value is the mean ± SE of 4 replicates. Treatment conditions are described in Fig. 1. At the same time point, numerical values with different letters are significantly different (*P *< 0.05).

Fig. 4 demonstrates the changes in approximated initial slope of the fluorescence transient (*M*_o_) and in the redox state of PSI expressed as (1-*V*_i_)/(1-*V*_j_). At normal temperature, spraying SA did not change *M*_o _and (1-*V*_i_)/(1-*V*_j_). After heat stress, *M*_o _and (1-*V*_i_)/(1-*V*_j_) rose rapidly. During recovery, *M*_o _and (1-*V*_i_)/(1-*V*_j_) of SA-treated leaves rapidly declined, and these parameters were markedly less in SA-treated leaves than in the control leaves on the first day of recovery (Day 3).

### PSII efficiency and excitation energy dissipation

**Figure 5:**
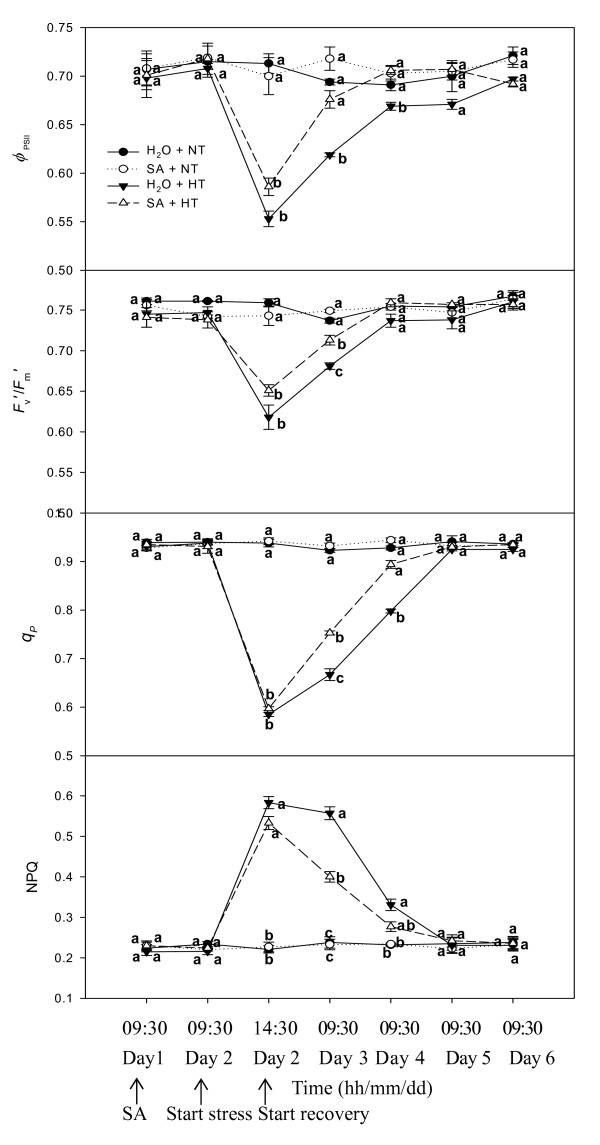
**PSII efficiency and excitation energy dissipation in leaves of grape plants sprayed with H_2_O (*filled circles*) and SA (*open circles*) at normal growth temperature (NT, 25°C), and treated with H_2_O (*filled triangles*) and SA (*open triangles*) under heat stress (HT, 43°C) and recovery**. Each value is the mean ± SE of 4 replicates. Treatment conditions are described in Fig. 1. At the same time point, numerical values with different letters are significantly different (*P *< 0.05).

PSII efficiency and excitation energy dissipation in grape leaves was examined by modulated fluorescence techniques. Fig. 5 shows that SA had no effect on the actual PSII efficiency (Φ_PSII_), the efficiency of excitation energy capture by open PSII reaction centers (*F*_v_'/*F*_m_'), the photochemical quenching coefficient (*q*_p_), or on non-photochemical quenching (NPQ) at the normal temperature. Heat stress led to a sharp decrease of *F*_v_'/*F*_m_', Φ_PSII _and *q*_p_, and a striking increase of NPQ irrespective of SA-treatment. With recovery, *F*_v_'/*F*_m_', Φ_PSII _and *q*_p _gradually rose; moreover, these parameters in SA-treated leaves were always greater than those in control leaves. Φ_PSII _values in SA-treated leaves were always significantly greater than in the control during recovery. On the first day of recovery (Day 3), NPQ of SA treatments declined rapidly, but NPQ of the controls remained higher. During the rest of the recovery period, there were no obvious differences in NPQ between SA treatments and the controls.

### Rubisco activation state

**Figure 6:**
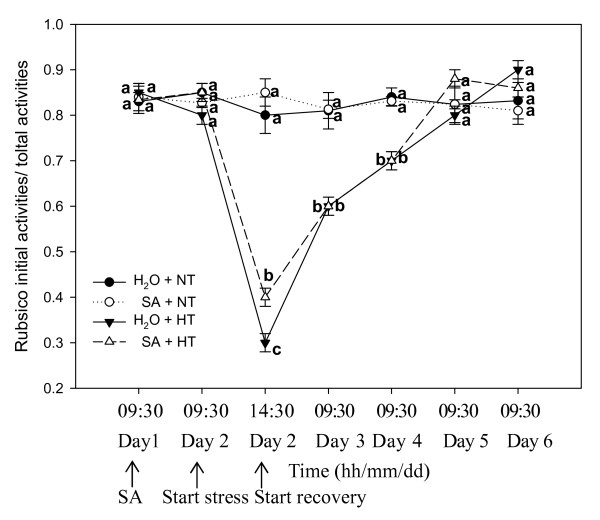
**Rubisco activation state in leaves of grape plants sprayed with H_2_O (*filled circles*) and SA (*open circles*) at normal growth temperature (NT, 25°C), and treated with H_2_O (*filled triangles*) and SA (*open triangles*) under heat stress (HT, 43°C) and recovery**. Each value is the mean ± SE of 4 replicates. Treatment conditions are described in Fig. 1. At the same time point, numerical values with different letters are significantly different (*P *< 0.05).

Fig. 6 demonstrates the changes in activation state of Rubisco (initial activities/total activities) in grape leaves. At normal temperatures, spraying SA did not change the ratio. In response to the heat stress, the ratio declined rapidly; however, SA-treated plants had a greater Rubisco activation state than the controls. During the recovery period, the Rubisco activation state of SA-treated leaves became similar to that of the non-stressed controls.

### HSP 21 in the chloroplast

**Figure 7:**
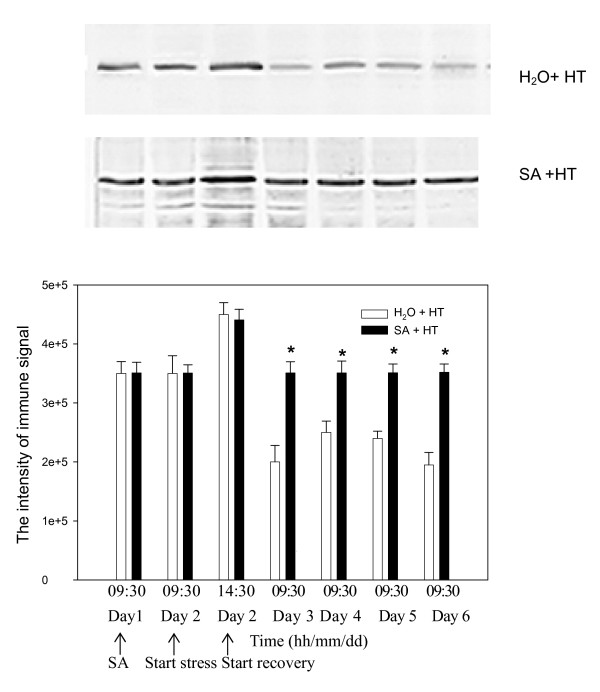
**HSP21 in leaves of grape plants sprayed by treated with H_2_O and SA under heat stress (HT, 43°C) and recovery**. Thylakoid membranes were extracted from leaves. Equal amounts (10 μg) of protein were subjected to SDS-PAGE and transferred to a nitrocellulose membrane. Thereafter, the membrane was incubated with anti-Arabidopsis thaliana HSP21 antibody. Treatment conditions are described in Fig. 1. * indicates a significant difference (*P *< 0.05) between the control and SA-treated plants at the same time point.

HSP21 is found only in the chloroplast, and a 21 kDa peptide was in the grape leaves (Fig.7) in both SA-pretreated and control leaves. SA did not significantly (*P *< 0.05) change the immune signal of HSP21 before heat stress. When SA-pretreated and control leaves were stressed, they both showed higher levels of the immune signal. However, during recovery, HSP21 levels in the SA-pretreatment remained high until the end of the experiment while those in the control decreased below pre-stress levels.

## Discussion

In this experiment, the *P*_n _of plants sprayed with H_2_O and maintained at normal temperatures was 6.48 ± 0.33 μmol m^-2 ^s^-1 ^at 14:30 h on Day 2 of the experiment, significantly (*P *< 0.05) higher than the *P*_n _of heat stressed plants sprayed with H_2_O or SA (Fig. 1). Therefore, the decrease of *P*_n _of SA-treated and control leaves under heat stress from 9:30 to 14:30 h on Day 2 was not due to a diurnal change in photosynthesis, but instead due to heat stress. SA did not alter *P*_n _significantly in plants maintained at the normal growth temperature, but it mitigated the decrease in *P*_n _under heat stress and promoted the increase in *P*_n _during recovery (Fig. 1). Under heat stress, change of *C*_i _was opposite to that of *P*_n _in the control and SA-treated leaves (Fig. 1), indicating that the decrease of *P*_n _under heat stress was due to non-stomatal factors. During recovery, the strong decrease in *C*_i _in control heat stressed plants (on Day 3) can be caused by the heat induced closing of stomata (less *g*_s_). Therefore, *g*_s _may have been a main constraint to *P*_n _for control plants at this time. But during the following recovery period, relative lower *P*_n _for control plants was not accompanied by lower *C*_i _and *g*_s_. SA treated leaves showed bigger *P*_n_, *C*_i _and *g*_s _after the first recovery day (Fig.1). These results may be related to electron transport and energy distribution. This can be seen by the changes in PSII parameters (Figs. 2, 3, 4 &5).

PSII is often considered the most heat-sensitive component of the photochemistry, and the oxygen-evolving complex within the PSII is very sensitive to heat stress [33]. Obviously, an increase in heat resistance of the oxygen-evolving complex would help increase the thermotolerance of PSII. Chlorophyll fluorescence parameters have been used to detect and quantify heat stress induced changes in PSII [34], and appearance of a K-step in the OJIP polyphasic fluorescence transient can be used as a specific indicator of injury to the oxygen-evolving complex [32]. In this study, we took advantage of the appearance of a K-step in the OJIP polyphasic fluoroscence transient to examine if SA-induced protection or improvement to PSII during heat stress and the recovery was related to the oxygen-evolving complex. W_K _in both control and SA treatments significantly increased when these plants were exposed to heat stress, but W_K _in the SA- treated plants dropped quickly while W_K _of the controls dropped slowly during recovery (Fig. 2). Therefore, the above hypothesis is supported by the data.

The PSII reaction center is also one of the sites damaged by heat stress [35]. Our results showed that the increased thermostability of PSII induced by SA treatment was partly associated with an increase in the thermostability of the PSII center. It was also observed that the density of Q_A_^- ^reducing PSII reaction centers in SA-treated plants increased more rapidly than in the controls during recovery from heat stress (Fig. 3). This was also confirmed by a quicker increase in SA-treated plants in *q*_p _(Fig.5) which can represent the fraction of open PSII reaction centers [36]. The results support the hypothesis that SA-induced protection of PSII during heat stress and the recovery was involved in several aspects of PSII function, such as the O_2_-evolving complex and the PSII reaction center.

In these experiments, the much lower *ψ*_Eo _and *ϕ*_Eo _showed that the activity of the electron transport beyond Q_A _was inhibited in heat stressed grape leaves (Fig. 2). The results indicated that heat stress also damaged the acceptor side of PSII. In addition, *ψ*_Eo _and *ϕ*_Eo _of SA-treated leaves increased more rapidly than that of the control leaves during recovery, indicating that SA can protect the acceptor side of PSII. In addition, the change in the ratio of (1-*V*_i_)/(1-*V*_j_) may suggest that SA also protected PSI, allowing more rapid recovery from heat stress (Fig.5).

Efficiency of PSII under steady-state irradiance (Φ_PSII_) is the product of *q*_p _and the efficiency of excitation capture *F*_v_'/*F*_m_' by open PSII reaction centers under non-photorespiratory conditions. Under heat stress, SA-treated and control leaves had much lower Φ_PSII _(Fig. 5), and had greater thermal dissipation of excitation energy as measured by increased NPQ (Fig. 5). With the recovery from heat stress, Φ_PSII _of SA-treated and control plants gradually increased, and this was accompanied by increases in *F*_v_'/*F*_m_' and *q*_p_, and a rapid decline of NPQ in SA-treatment. However, NPQ of control plants slowly declined. In addition, *P*_n _of SA-treated plants was greater than that of the control plants. This indicated that during recovery SA-treated plants do not need to dissipate much energy as heat, but instead are able to convert more energy into electron transport.

Inhibition of photosynthesis by heat stress has long been attributed to an impairment of electron transport [37]. However, other studies support the idea that the initial site of inhibition is associated with a Calvin cycle reaction, specifically the inactivation of Rubisco [38]. Measurements of the activation state of Rubisco in leaves, determined from the ratio of initial extractable activity to the activity after incubation under conditions that fully carbamylate the enzyme, show that the activation state of Rubisco decreases when net photosynthesis is inhibited by heat stress [39]. Here, under heat stress Ribisco activation state was greater in SA treated leaves than in the controls (Fig. 6), indicating that SA may alleviate Rubisco inactiviation under heat stress. However, SA treatment did nothing to improve the rate of recovery of the Rubisco activation state.

Evidence suggests that the small chloroplast heat-shock protein (HSP21) is involved in plant thermotolerance, and protects the thermolabile PS II and whole-chain electron transport [12,40]. HSPs including HSP21 have a high capacity to bind, stabilize and prevent protein aggregation, and help them regain normal function following stress [41]. In this study, HSP 21 levels increased in both SA-treated and control leaves during heat stress (Fig.7). Under severe heat stress, many proteins in the chloroplast are subject to denaturation, and HSPs function as molecular chaperones to provide protection. When stressed plants recover, HSPs are no longer made, and further degraded [42]; but, here in controls the levels of HSP21 decreased during the recovery to below initial levels (Fig.7). Similarly, Park et al [43] also reported that HSP18 levels in creeping bentgrass during recovery were lower than initially. However, SA treatment here maintained HSP21 at high levels in the recovery period. These data indicate that SA may alleviate Rubisco deactivation as well as enhance PSII recovery through HSP21.

## Conclusions

SA pretreatment did not significantly influence photosynthesis of grape leaves at normal growth temperatures. However, SA pretreatment alleviated the decrease of *P*_n _under heat stress, apparently in part through maintaining a higher Rubisco activation state and greater PSII efficiency. SA also accelerated the increase of *P*_n _mainly through the more rapid recovery of PSII function after heat stress. These SA effects may be related to higher levels of HSP21. Other mechanisms by which SA protects photosynthesis in grape leaves are still to be determined.

## Methods

### Plant materials and treatments

Stem cuttings of grape (*Vitis vinifera *L.) 'Jingxiu' were rooted in the pots containing a mixture of 4 peatmoss: 6 perlite (V/V) and grown in a greenhouse under mist conditions. When the cuttings were rooted, they were repotted into larger pots, grown for about 10 weeks in a greenhouse at 70-80% relative humidity, 25/18°C day/night cycle, and with the maximum photosynthetically active radiation at about 1,000 μmol m^-2 ^s^-1^.

Young grape plants with identical growth (10 leaves) were acclimated for two days in a controlled environment room (70 - 80% relative humidity, 25/18°C day/night cycle and 800 μmol m^-2 ^s^-1^) and divided into two groups. On the following day (the first day of the experiment, Day 1), chlorophyll fluorescence and gas exchange parameters were analyzed at 9:30 h for all plants. One group of plants was then sprayed with 100 μM SA solution, and the other group was sprayed with water. On Day 2, the same parameters were measured at 9:30 h. Half of the SA-treated and H_2_O-treated plants were then heat stressed at 43°C until 14:30 h; the other half remained at 25°C until 14:30 h. Relative photosynthesis parameters were then rapidly measured. The stressed plants were then allowed to recover at 25°C. Chlorophyll florescence and gas exchange parameters were measured at 9:30 h each day during the following four days of recovery (Day 3, Day 4, Day 5 and Day 6). All of the above measurements were made on the fifth leaf from the top of each plant. Four replications were made with leaves from different grape plants.

### Analysis of photosynthetic gas exchange

Photosynthetic gas exchange was analyzed with a Li-Cor 6400 portable photosynthesis system which can control photosynthesis by means of photosynthetic photon flux density (PPFD), leaf temperature and CO_2 _co-ncentration in the cuvette. Net photosynthetic rate (*P*_n_), stomatal conductance (*g*_s_) and substomatal CO_2 _concentration (*C*_i_) were determined at a concentration of ambient CO_2 _(360 μmol mol^-1^) and a PPFD of 800 μmol m^-2^s^-1^.

### Analysis of chlorophyll fluorescence

Chlorophyll fluorescence was measured with a FM-2 Pulse-modulated Fluorimeter (Hansatech, UK). The maximal fluorescence level in the dark-adapted state (*F*_m_) were measured by a 0.8 s saturating pulse at 8000 μmol m^-2 ^s^-1 ^after 20 min of dark adaptation. When measuring the induction, the actinic light was offered by the FMS-2 light source. The steady-state fluorescence (*F*_s_) was thereafter recorded and a second 0.8 s saturating light of 8000 μmol m^-2^s^-1 ^was given to determine the maximum fluorescence in the light-adapted state (*F*_m_'). The actinic light was then turned off; the minimal fluorescence in the light-adapted state (*F*_o_') was determined by illumination with 3 s of far red light. The following parameters were then calculated: (1) efficiency of excitation energy captured by open PSII reaction centers, *F*_v_'/*F*_m_'= (*F*_m_' - *F*_o_')/*F*_m_'; (2) the photochemical quenching coefficient, *q*_p _= (*F*_m_' - *F*_s_)/(*F*_m_' - *F*_o_'); (3) the actual PSII efficiency, Φ_PSII _= (*F*_m_' - *F*_s_)/*F*_m_'; and (4) non-photochemical quenching, NPQ = *F*_m_/*F*_m_' - 1[44].

### Measurement of the polyphasic transient of chlorophyll a fluorescence (OJIP test)

The so-called OJIP-test was employed to analyze each chlorophyll a fluorescence transient by a Handy Plant Efficiency Analyzer (PEA, Hansatech, UK), which could provide information on photochemical activity of PSII and status of the plastoquinone pool [45]. Before measurement, leaves were dark-acclimated for 20 minutes. The transients were induced by red light of about 3000 μmol photons m^-2 ^s^-1 ^provided by an array of six light emitting diodes (peak 650 nm). The fluorescence signals were recorded within a time span from 10 μs to 1 s with a data acquisition rate of 10 μs for the first 2 ms and every 1 ms thereafter. The fluorescence signal at 50 μs was considered as a true *F*_o_. The following data from the original measurements were used: maximal fluorescence intensity (*F*_m_); fluorescence intensity at 300 μs (*F*_k_) [required for calculation of the initial slope (*M*_o_) of the relative variable fluorescence (V) kinetics and *W*_k_]; and the fluorescence intensity at 2 ms (the J-step) denoted as *F*_j_, the fluorescence intensity at 30 ms (the I-step) denoted as *F*_i_. Terms and formulae are as follows: a parameter which represent the damage to oxygen evolving complex (OEC), *W*_k _= (*F*_k _- *F*_o_)/*F*_j _- *F*_o_); approximated initial slope of the fluorescence transient, *M*_o _= 4(*F*_k _- *F*_o_)/(*F*_m _- *F*_o_); probability that a trapped exciton moves an electron into the electron transport chain beyond Q_A_^-^, *ψ*_Eo _= *ET*_o_/TR_o _= (*F*_m _- *F*_j_)/(*F*_m _- *F*_o_); quantum yield for electron transport (at t = 0), Φ_Eo _= *ET*_o_/ABS = [1 - (*F*_o_/*F*_m_)] × *ψ*_Eo_; and the density of Q_A_-reducing reaction centers, RC_QA _= *ϕ*_Po _× (V_j_/*M*_o_) × (ABS/CS). The formulae in Table 1 illustrate how each of the above-mentioned biophysical parameters can be calculated from the original fluorescence measurements.

**Table 1:** Summary of parameters, formulae and their description using data extracted from chlorophyll a fluorescence (OJIP) transient.

Fluorescence parameters	Description
*F*_t_	Fluorescence intensity at time t after onset of actinic illumination
*F*_50 μs_	Minimum reliable recorded fluorescence at 50 μs with the PEA fluorimeter
*F*_k _(F_300 μs_)	Fluorescence intensity at 300 μs
*F*_P_	Maximum recorded (= maximum possible) fluorescence at P-step
Area	Total complementary area between fluorescence induction curve and F = Fm
ABS	Absorption of energy
TR	Trap of energy
CS	Excited Cross section
Derived parameters (Selected OJIP parameters)	
*F*_o_≅*F*_50 μs_	Minimum fluorescence, when all PSII RCs are open
*F*_m _= *F*_P_	Maximum fluorescence, when all PSII RCs are closed
*V*_j _= (*F*_2 ms _-- *F*_o_)/(*F*_m _-- *F*_o_)	Relative variable fluorescence at the J-step (2 ms)
*V*_i _= (*F*_30 ms _-- *F*_o_)/(*F*_m _-- *F*_o_)	Relative variable fluorescence at the I-step (30 ms)
W_K_= (*F*_300 μs_-- *F*_o_/(F_j_-- *F*_o_)	Represent the damage to oxygen evolving complex OEC
*M*_o _= 4 (*F*_300 μs _-- *F*_o_)/(*F*_m_-- *F*_o_)	Approximated initial slope of the fluorescence transient
Yields or flux ratios	
*ϕ*_Po _= TR_o_/ABS = 1-- (*F*_o_/*F*_m_) = *F*_v_/*F*_m_	Maximum quantum yield of primary photochemistry at t = 0
*ϕ*_Eo _= ET_o_/ABS = (*F*_v_/*F*_m_) × (1 -- *V*_j_)	Quantum yield for electron transport at t = 0
*ψ*_Eo _= ET_o_/TR_o _= 1 -- *V*_j_	Probability (at time 0) that a trapped exciton moves an electron into the electron transport chain beyond Q_A_^-^
*δ*_Ro _= (1 -- *V*_i_)/(1 -- *V*_j_)	Efficiency with which an electron can move from the reduced intersystem, electron acceptors to the PSI end electron acceptors
Density of reaction centers. RC_QA _= *ϕ*_Po _× (ABS/CS_m_) × (*V*_j_/*M*_o_)	Amount of active PSII RCs (QA-reducing PSII reaction centers) per CS at t = m

### Extraction and assay of Ribulose-1,5-bisphosphate carboxylase/oxygenase (Rubisco, EC4.1.1.39)

Leaves disks (1 cm^2 ^each) were taken, then frozen in liquid nitrogen, and stored at -80°C until assay. Rubisco was extracted according to Chen and Cheng [46]. Three frozen leaf disks were ground with a pre-cooled mortar and pestle in 1.5 mL extraction buffer containing 50 mM Hepes-KOH (pH7.5), 10 mM MgCl_2_, 2 mM EDTA, 10 mM dithiothreitol (DDT), 1% (v/v) Triton X-100, 1% (w/v) bovine serum albumin (BSA), 10% (v/v) glycerol, 0.5 mM phenylmethylsulfonyl fluoride (PMSF), and 5% (w/v) insoluble polyvinylpolypyrrolidone (PVPP). The extract was centrifuged at 13 000 × g for 5 min in an Eppendorf microcentrifuge at 4°C, and the supernatant was used immediately for enzyme assays.

For Rubisco initial activity, a 50 μl sample extract was added to a semi-microcuvette containing 900 μl of an assay solution, immediately followed by adding 50 μl 0.5 mM RuBP, mixing well. The change of absorbance at 340 nm was monitored for 40 s. For Rubisco total activity, 50 μl 0.5 mM RuBP was added 15 min after a sample extract was combined with assay solution to activate all the Rubisco fully. Rubisco activation state was calculated as the ratio of initial activity to total activity [46,47].

### Tissue fractionation and western blot analysis for heat shock proteins (HSP21)

Total protein was extracted according to the methods of Hong et al. [48] with some modification. Leaves were immediately frozen in liquid nitrogen and homogenized 1:3 (w/v) in 150 mM Tris buffer, pH 7.8, containing 2 mM EDTA-Na_2_, 10 mM ascorbic acid, 10 mM MgCl_2_, 1 mM PMSF, 0.2% (v/v) 2-mercaptoethanol, 2% (w/v) PVPP and 2% (w/v) SDS. Protein extracts were centrifuged at 12 000 × g for 15 min and the procedure repeated twice.

For western blot analysis, SDS-PAGE was carried out in 10% (v/v) acrylamide slab gels, the samples were diluted with an equal volume of buffer and heated at 100°C for 5 min, then centrifuged at 10,000 × g for 10 min. Polypeptides were separated using Bio-Rad Miniprotean II slab cell. Electrophoretic transfer of polypeptides from SDS polyacrylamide gels to nitrocellulose membranes (0.45 mm, Amersham Life Science) was conducted in 25 mM Tris (pH 8.3), 192 mM glycine and 20% (w/v) methanol. After rinsing in TBS buffer (10 mM Tris-HCl, pH 7.5, 150 mM NaCl), the membranes were preincubated for 2 h at room temperature in a blocking buffer containing 1% (w/v) bovine serum albumin (BSA) dissolved in TBST [TBS, 0.05% (v/v) Tween 20]. They were then incubated with gentle shaking for 2 h at room temperature in *Arabidopsis *anti-HSP21 antibody (Agrisera Company, Sweden). Following extensive washes with TBST buffer, the membranes were incubated with goat antirabbit IgG-alkaline phosphatase conjugate (1:1000 diluted in TBST) at room temperature for 1 h, and were then washed with TBST. The locations of antigenic proteins were visualized by incubating the membranes with 5-bromo-4-chloro-3-indolyl. Protein concentrations were determined by the method of Bradford [49] with BSA as a standard.

### Statistical analyses

Data were processed with SPSS 13.0 for Windows, and each mean and standard error in the figures represents four replicate measurements. Differences were considered significant at a probability level of *P *< 0.05.

## Abbreviations

*C*_i_: substomatal CO_2 _concentration; *F*_o_' and *F*_m_': the minimal and maximum fluorescence in the light-adapted state; *F*_s_: the steady-state fluorescence; *F*_v_'/*F*_m_': efficiency of excitation energy capture by open PSII reaction centers; HSP: heat shock protein; NPQ: non-photochemical quenching; OEC: oxygen evolving complex; *P*_n_: net photosynthetic rate; PSII: photosystem II; RC_QA_: density of QA -reducing reaction centers; Rubisco: ribulose bisphosphate carboxylase/oxygenase; *q*_p_: photochemical quenching coefficient; RuBP: ribulose-1,5- bisphosphate; SA: salicylic acid; Φ_PSII_: actual PSII efficiency; *M*_o_: approximated initial slope of the fluorescence transient; *ψ*_Eo_: probability that a trapped exciton moves an electron into the electron transport chain beyond QA^-^; *ϕ*_Eo_: quantum yield for electron transport.

## Authors' contributions

WLJ designed the experiments, performed a part of the experiments and wrote the manuscript. FL performed a part of the experiments. LW helped design the experiment and reviewed the manuscript. DW helped design the experiment. LGJ helped design the experiment. CJS and LHB helped in measuring CO_2 _assimilation and chlorophyll a fluorescence. LSH directed the study. All authors have read and approved the final manuscript.
